# Transcranial Magnetic Stimulation of Posterior Parietal Cortex Modulates Line-Length Estimation but Not Illusory Depth Perception

**DOI:** 10.3389/fpsyg.2019.01169

**Published:** 2019-05-22

**Authors:** Adriana Salatino, Gaetana Chillemi, Federica Gontero, Marisa Poncini, Maria Pyasik, Anna Berti, Raffaella Ricci

**Affiliations:** ^1^SpAtial, Motor and Bodily Awareness Research Group, Department of Psychology, University of Turin, Turin, Italy; ^2^IRCCS Centro Neurolesi “Bonino Pulejo,” Messina, Italy; ^3^Neuroscience Institute of Turin, Turin, Italy

**Keywords:** rTMS, necker cube, posterior parietal cortex, landmark task, visuospatial attention

## Abstract

Transcranial Magnetic Stimulation (TMS) may affect attentional processing when applied to the right posterior parietal cortex (PPC) of healthy participants in line with neuropsychological and neuroimaging evidence on the neural bases of this cognitive function. Specifically, the application of TMS to right PPC induces a rightward attentional bias on line length estimation in healthy participants (i.e., neglect-like bias), mimicking the rightward bias shown by patients with unilateral spatial neglect after damage of the right PPC. With the present study, we investigated whether right PPC might play a crucial role in attentional processing of illusory depth perception, given the evidence that a rightward bias may be observed in patients with neglect during perception of the Necker Cube (NC). To this end, we investigated the effects of low-frequency rTMS applied to the right or left PPC on attentional disambiguation of the NC in two groups of healthy participants. To control for the effectiveness of TMS on visuospatial attention, rTMS effects were also assessed on a frequently used line length estimation (i.e., the Landmark Task or LT). Both groups also received sham stimulation. RTMS of the right or left PPC did not affect NC perception. On the other hand, rTMS of the right PPC (but not left PPC) induces neglect-like bias on the LT, in line with previous studies. These findings confirm that right PPC is involved in deployment of spatial attention on line length estimation. Interestingly, they suggest that this brain region does not critically contribute to deployment of visuospatial attention during attentional disambiguation of the Necker Cube. Future investigations, targeting different areas of fronto-parietal circuits, are necessary to further explore the neuro-functional bases of attentional contribution to illusory depth perception.

## Introduction

Transcranial magnetic stimulation (TMS) may be used in healthy volunteers to transiently interfere with the activity of a focal brain region and test its contribution to the occurrence of a motor, perceptual, or cognitive event (Pascual-Leone et al., 1991; Miniussi et al., 2013). In the last decades, this approach has been employed to investigate the role of the posterior parietal cortex (PPC) in visuospatial attention (Fierro et al., 2000, 2001, 2006; Brighina et al., 2002; Ellison et al., 2004; Bjoertomt et al., 2009; Ricci et al., 2012; Salatino et al., 2014; Giglia et al., 2015; Giglhuber et al., 2017). Most of these studies have shown that the application of online single pulse TMS or high-frequency repetitive TMS (rTMS) (Fierro et al., 2000, 2001, 2006; Brighina et al., 2002; Ellison et al., 2004; Bjoertomt et al., 2009; Ricci et al., 2012; Salatino et al., 2014; Giglia et al., 2015; Giglhuber et al., 2017) and, in one study, offline low-frequency rTMS (Bagattini et al., 2015) to the right PPC induces, in healthy participants, an attentional bias on line length-estimation that mimics the rightward bias typically observed in patients with unilateral visuospatial neglect (Bisiach et al., 1998).

Visuospatial neglect refers to inability of patients to explore the side of space contralateral to a brain lesion – most often of the right hemisphere – and to attend to contents of this portion of space (Heilman et al., 1993; Vallar and Ronchi, 2006; Ricci et al., 2016). Anatomo-clinical correlation studies show a predominant role of right PPC in neglect syndrome (Vallar, 2001; Marshall et al., 2002; Mort et al., 2003), although other areas functionally connected to PPC – i.e., frontal and subcortical structures, and superior temporal gyrus (Karnath et al., 2001, 2004) – can be implicated in neglect symptomatology (Hillis, 2006). When patients with visuospatial neglect are asked to bisect horizontal lines or judge the length of two segments composing pre-bisected lines, they bisect lines toward the ipsilesional space or judge the ipsilesional segment as longer, respectively. Neglect behavior is not explained by deficits of elementary or intermediate vision (Driver et al., 1992; Ricci et al., 1999, 2000; Ricci and Chatterjee, 2001), but rather it is ascribed to disruption of higher level attentional processes (Heilman et al., 1993; Bisiach and Berti, 1995). In line with this interpretation, Bisiach et al. (1999) have shown that pathological constraints imposed upon spatial attention by the brain lesion, in patients with neglect, affected perception of illusory three-dimensional stimuli (i.e., the Necker cubes, NC). Interestingly, altered perception of the NC in patients with neglect was positively correlated with rightward perceptual and response biases on the Landmark Task (Bisiach et al., 1999). Early perceptual processing of two-dimensional visual stimuli, as the NC, leads to interpretation of those configurations as three-dimensional objects that are compatible with either of two contrasting perspectives. Based on their findings, Bisiach et al. (1999) proposed that perspective disambiguation of the Necker cube is carried out by the dynamics of a complex attentional vector, which is bent to the right in patients with left-neglect.

The Necker cubes (Necker, 1832) are ambiguous figures, that provide compelling examples of how the visual system ensures perceptual organization and stability. Previous evidence has shown that the perceptual reversal rate can be influenced by several factors, such as, for example, figure size (Washburn et al., 1931; Spitz and Lipman, 1962), luminance (Mull et al., 1954; Heath et al., 1963), observation time (Brown, 1955) and intermittent presentation (Orbach et al., 1963; Magnussen, 1972). In addition, it is not prevented by the absence of retinal image motion (Pritchard, 1958; Evans and Marsden, 1966; Gregory, 1970), and is not accompanied by specific eye movements (Flamm and Bergum, 1977). In an early study changes in the apparent distance of a vertex of the Necker cube were associated with pupillometric changes similar to those observed with changes in real depth (Enright, 1987). It was also found that if a Necker cube is tachistoscopically presented, so that one of the two central vertices coincides with the fixation point, that vertex is perceived as nearer (Kawabata et al., 1978; Kawabata, 1986). In the last 10 years, neurophysiological EEG studies have demonstrated momentary fluctuations of brain activity during perception of the Necker cube in right inferior parietal cortex (Britz et al., 2009) and between occipital and frontal areas (Shimaoka et al., 2010). In addition, an fMRI study (Inui et al., 2000) has shown bilateral (symmetrical) activations of premotor and parietal areas associated with NC perception, similar to those that occur during mental image manipulation (Inui et al., 2000).

Transcranial Magnetic Stimulation studies are consistent with neglect neuroanatomy in showing the relevance of right hemisphere PPC in the orienting of spatial attention on line length estimation. However, to our knowledge, no TMS studies have been conducted to investigate the contribution of PPC to the dynamics of spatial attention in Necker cube perception. Thus, in the present study, we aimed at investigating whether rTMS of the right PPC might affect Necker cubes disambiguation in healthy participants, similarly to what occur in patients with left-neglect. The effects of rTMS applied to PPC were also assessed on a line length estimation task, i.e., the Landmark Task (LT), given the evidence that: (1) attentional biases on the NC and the Landmark tasks were found to correlate in patients with left-neglect (2) neglect-like bias on the Landmark task has been consistently induced by application of TMS to right PPC in healthy participants. We hypothesize that active rTMS of the right PPC, but not of left PPC or sham stimulation, would have induced a rightward attentional bias on both LT and NC, similar to those observed in patients with left-neglect.

## Materials and Methods

The effects of inhibitory rTMS of PPC on Necker cube perception (NC) and LT were assessed in twenty-six healthy participants. RTMS was applied to right PPC in Experiment 1 (*n* = 13) and to left PPC in Experiment 2 (*n* = 13). Tasks were performed before and after rTMS.

### Experiment 1

#### Participants

Thirteen right-handed healthy volunteers (9 women; mean age 26.77 years, *SD* = 6.65) participated in the first Experiment, which was composed of two studies. All participants had normal vision, and no history of neurological or psychiatric illness.

All participants were screened against inclusion/exclusion criteria for a safety use of TMS (Rossi et al., 2009). Participants were given a detailed explanation of the procedure, and they gave written informed consent to participate in the study, that was approved by the Ethical Committee of the University of Turin. Participants underwent rTMS of the Right PPC (R PPC). A sham session, with the coil placed tangentially to the Right PPC, was also administered as control condition.

##### Study 1: landmark task

For the first study, the effects of rTMS were assessed on the Landmark Task (LT, Bisiach et al., 1998). The LT was administered before (baseline condition) and soon after the intervention. As control condition, the participants received sham stimulation, in the same day of active rTMS, with an interval of about 1 h between sessions. As for active rTMS, sham rTMS, the tasks were administered before and after the intervention. The order between active and sham rTMS was balanced between participants.

##### Study 2: necker cube

For the second study, the effects of rTMS were assessed on the Necker Cube (NC), a task previously employed in patients with neglect (Bisiach et al., 1999). As control condition, participants underwent a sham session, in the same day of the active rTMS, with an interval of about 1 h between sessions. For both sessions, the task was administered before (baseline condition) and after the interventions and the order between active and sham rTMS was balanced between participants.

Between the two studies, there were an interval of at least 1 week.

#### Magnetic Stimulation

Transcranial Magnetic Stimulation was performed with a Magstim Super Rapid 2 stimulator (Magstim, Whitlan, Dyfed, Wales, United Kingdom) connected to a 70-mm figure-of-eight coil, using a computer-assisted system able to deliver the TMS pulse time-locked to the visual stimulus. The handle of the coil pointed backward and 45° downward from the parasagittal line. The inter-stimulus interval had a duration of at least 4 s. Participants’ rMT was defined as the lowest stimulus intensity able to elicit a visible twitch in the abductor pollicis brevis muscle of the right hand in at least 5 of 10 consecutive stimulations of the motor hotspot (mean ± SD 54.15 ± 5.54% of the maximum stimulator output). They were comfortably seated in front of a computer screen, which was centered on their sagittal mid-plane, at a distance of 60 cm from the screen.

#### Hunting Procedure

This procedure is a new site-finding TMS protocol to easily identify the optimum parietal location, or “hot spot,” where the TMS may modulate visuospatial perception on a line length estimation task (the LT). Single-pulse TMS at 115% of participants’ resting motor threshold was applied 150 ms after the visual stimulus onset over nine different sites of a 3 cm × 3 cm grid, centered over right (Experiment 1) or left PPC (Experiment 2), over P4 and P3 according to the 10–20 EEG system, respectively. The coil was moved for each spot of the grid until the more sensitive spot was found (for details see Salatino et al., 2014).

#### Visual Stimuli

**FIGURE 1:**
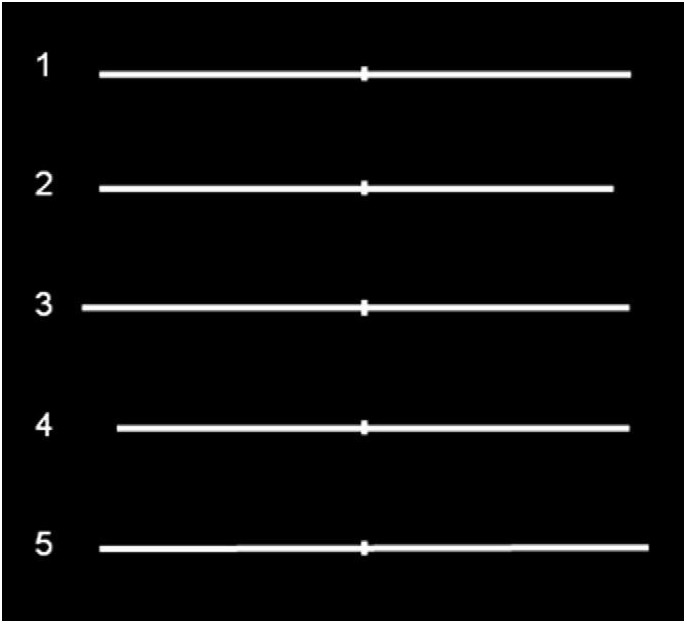
Symmetrically and asymmetrically bisected lines used in the study. Line 1 (symmetrically bisected): left segment 10.13° of visual angle/right segment 10.13°; Line 2 (left-elongated): left segment 10.13°/right segment 9.47°; Line 3 (left-elongated): left segment 10.79°/right segment 10.13°; Line 4 (right-elongated): left segment 9.47°/right segment 10.13°; Line 5 (right-elongated): left segment 10.13°/right segment 10.79°. Figure adapted with permission from Ricci et al. (2012).

For Study 1 (i.e., the *Landmark Task*), the visual stimuli were five different types of white horizontal lines with variable length from 195 to 210 mm, 0.09° of visual angle thick, previously divided into two parts by a vertical bar (10 mm long and 1 mm wide). Of the five lines, one was symmetrically transected in half, two had the bisecting shifted to the left compared to the objective middle point, and the other two, on the contrary, to the right, and were presented at the center of the black screen of a computer monitor for 150 ms (see Figure 1). Before each stimulus, a vertical line (0.95° of visual angle high) was presented for 500 ms at the center of the screen, to indicate the central fixation point. Subjects were required to report which segment composing the pre-bisected line was shortest (Task 1) and longest (Task 2), by pressing a button and responding as quickly as they could, without sacrificing accuracy for speed. The tasks were administered before and after 10 min (600 pulses) rTMS or sham session over right PPC. Within each task, each of three types of stimuli (line bisected on the left, right, or exactly in the center), were randomly presented 10 times (5 each for each line bisected on the left and right, and 10 for the line perfectly bisected in half), for 30 trials in total for each Task. Half of the subjects executed Task 1 first and then Task 2, and vice versa the other half.

**FIGURE 2:**
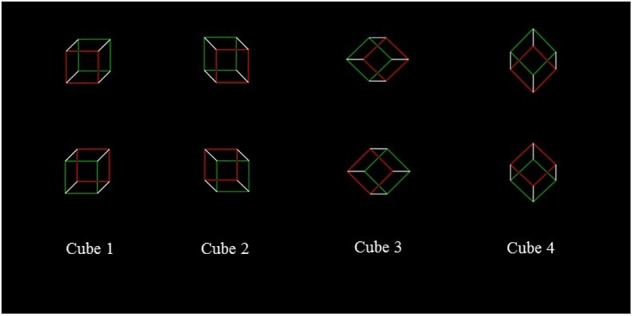
Necker cubes.

For Study 2 (i.e., the *Necker Cube*), we used eight different cubes, shown in four different orientations (see Figure 2). The edges of the two faces on the frontal plane were red or green, with the same brightness gradient; the remaining sides were white, and all the points of intersection were black, as well as the background. The side of the frontal surfaces measured 55 mm, and was 1 mm thick. Each stimulus was presented at the center of the computer screen. Participants were required to perform two tasks, reporting the color (green or red) of the face that, at first sight, they perceived as closest (“nearest face” – Task 3) and of the one that, at first sight, seemed most distant (“farthest face” – Task 4) by pressing two different buttons (which were codified as left vs. right for the data analysis). For each task, each stimulus was presented 10 times, for a total of 80 cubes, with a random order of presentation. Each cube was presented for 800 msec, after the presentation, for 500 msec, of a cross at the center of the screen, to indicate the central fixation point. Half of the subjects executed Task 3 first and then Task 4, and vice versa the other half. As for the LT, the NC Task was administered twice, before (baseline condition) and after 10 min rTMS or sham over right PPC.

### Experiment 2

#### Participants

A different sample of thirteen healthy volunteers (9 women; mean age 25.77 years, *SD* = 5.78) participated in Experiment 2, which was composed of two studies. As for the first Experiment, all participants were right-handers, had normal vision, and no history of neurological or psychiatric illness, and they underwent low-frequency rTMS applied at 90 of the rMT (mean ± SD 57.46 ± 5.62% of the maximum stimulator output) over left PPC (lPPC). A sham session, with coil placed tangentially to the scalp over the left PPC, was administered as control condition. Inclusion criteria were the same as in Experiment 1, and they gave their written informed consent to participate in the study, according to the Declaration of Helsinki and the Ethical Committee of the University of Turin.

The experiment method and stimuli were the same as Experiment 1, with the difference that the rTMS was applied to a control site, the left PPC. As for the first Experiment, Experiment 2 was composed of 2 studies: *Landmark Task* and *Necker Cube*. The procedures, as well as the visual stimuli, were identical to those in Experiment 1.

### Data Analysis

For both Experiments, when at least one variable in each analysis violated the criteria of normal distribution (*Shapiro–Wilk test*), non-parametric tests were used, and when multiple comparisons were present, the *p*-values were Bonferroni corrected. Effect sizes were estimated using the Pearson’s correlation coefficient r.

#### Landmark Task

For both Experiments, in order to analyze the overall participants’ performance, participants’ responses on the “*shorter task*” (i.e., subjects were required to report the “shorter’ segment composing the pre-bisected line, Task 1) were converted into “*longer task*” responses, (i.e., in subjects were required to report the “longer” segment composing the pre-bisected Task 2). Thus, data from the two opposite conditions were pooled together. Then, participants’ accuracy (i.e., the number of correct responses according to the task) for the asymmetrically bisected lines and “right” choices for the symmetrically bisected lines, expressed in percentages, were analyzed separately using the Wilcoxon test for dependent samples. The test was performed to compare the factors: TIME (Pre vs. Post) and rTMS (Active vs. Sham) as within subjects factors.

#### Necker Cube

In both Experiments, due to the different perspectives, the two frontal surfaces of cubes 1, 2 and 3 are horizontally separated with respect to one another, so that one surface is shifted to the “left” (hereafter indicated as the “left surface,” i.e., the surface bordered in red in the cube shown in the left upper corner of Figure 2) and the other one is shifted to the “right” (hereafter indicated as the “right surface,” i.e., the surface bordered in green in the same cube). Differently, for the cube 4, the frontal surfaces are vertically separated with respect to one another so that one surface is shifted upward (hereafter indicated as the “upper surface,” i.e., the surface bordered in green in the cube shown in the right upper corner of Figure 2), and the other one is shifted downward (hereafter indicated as the “lower surface,” i.e., the surface bordered in red in the same cube). In other words, according to Bisiach et al. (1999) it is possible to “assume that, after an early perceptual processing presents the flat configuration of a Necker cube as a three-dimensional object compatible with either of two contrasting perspectives, the disambiguation of the cube is carried out by the dynamics of a complex attentional vector” (page 137) and, “the first surface on which the vector impacts is interpreted as a surface external to the cube” (page 137). In line with this hypothesis, on cubes 1, 2, and 3, the vector impacts on the surface that is horizontally separated with respect to one another, while with cube 4, it impacts on the upper frontal surface, which is vertically separated with respect to one another. Following Bisiach et al. (1999) original work, the analysis was conducted pulling together the number of “nearest” responses given by participants to “left” surfaces with cubes 1, 2, and 3. On cube 4, the analysis was conducted on the total number of “nearest” responses given to the “upper” surface.

As for LT, for the NC results of the two complementary Tasks (Task 3 and Task 4) were pulled together. Then, participants’ responses, expressed in percentages, were analyzed for cubes 1+2+3 and for the cube 4, separately using the Wilcoxon test for dependent samples. The test was performed to compare the factors: TIME (Pre vs. Post) and rTMS (Real vs. Sham) as within subjects factors. The cumulative percentages concerning Task 3 + Task 4, are reported in Table 2 according to Group and conditions.

## Results

### Experiment 1

#### Landmark Task

**FIGURE 3:**
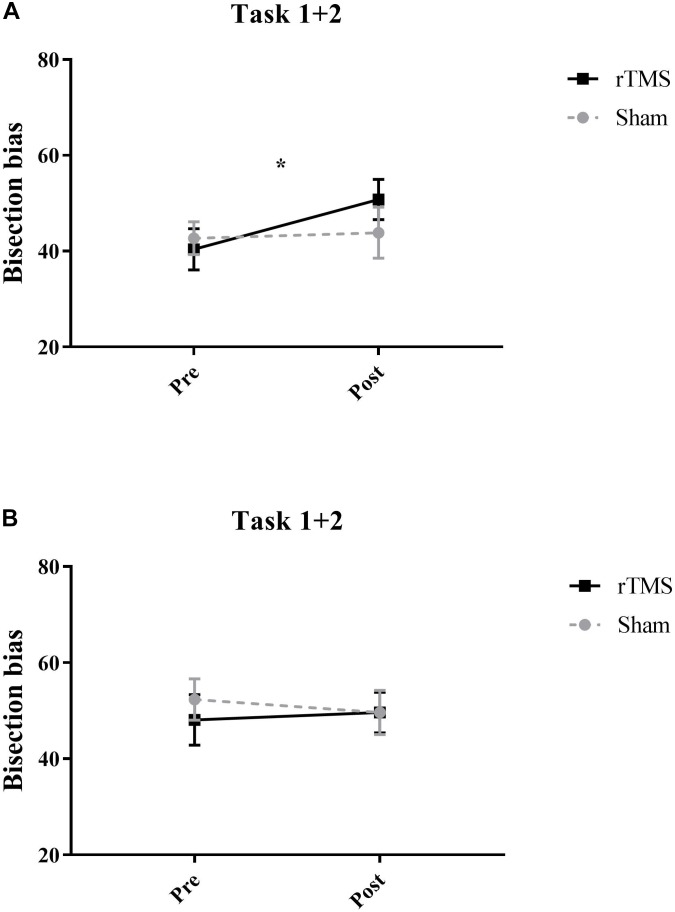
Mean participants’ scores pooled across tasks on the symmetrically bisected lines for Experiment 1 **(A)** and for Experiment 2 **(B)**. After active rTMS of the Right PPC, participants showed a significant rightward bias (significant at ^∗^*p* = 0.02) (i.e., left segment underestimation) in the symmetrical stimuli. Error bars represent standard error of means.

For the LT participants’, cumulative percentages concerning Task 1 + Task 2 are reported in Table 1 for the different conditions. Participants showed high accuracy (>90%) on the asymmetric stimuli, and no significant differences were found across conditions. The most interesting result relates to subjects’ performance on the symmetrically bisected lines. In those lines, indeed, after rTMS over the Right PPC, participants tended to more often choose the right segment as longer (50.8%) with respect to the baseline (40.4%). Thus, after the inference with the Right PPC, participants showed a significant rightward bias (mean = 40.3 ± 115.4; *Z* = 2.27, *p* = 0.02, *r* = -0.45) (i.e., left segment underestimation) similarly to the perceptual bias shown on this task by patients with left neglect, with respect to baseline trials. No significant effects were found for sham condition (see Table 1 and Figure 3A).

**Table 1:** Mean percentages (and SDs) of right choices as a function of site (A, Experiment 1, Right PPC; B, Experiment 2, Left PPC), and rTMS (Baseline, rTMS/Sham) for Symmetrically (Sym) bisected lines and global accuracy for the Asymmetrically bisected lines (Asym Acc).

A. Experiment 1, Right PPC					B. Experiment 2, Left PPC			
	
	Baseline	rTMS	Baseline	Sham	Baseline	rTMS	Baseline	Sham
Sym	40.4 (15.4)	50.8^∗^ (16.2)	42.7 (12.3)	43.8 (19.2)	48 (18.9)	49.6 (15.2)	52.3 (15.4)	49.6 (16.7)
Asym (Acc)	91.9 (6.8)	90.1 (4.4)	88.5 (5.8)	88 (7.1)	86.9 (6.4)	88.6 (7.4)	89.4 (5.7)	89 (7.7)


*^∗^Significantly different from Baseline.*

#### Necker Cube

The participants’ cumulative percentages concerning cubes 1, 2, and 3 and for the cube 4, are reported in Table 2 for the different conditions. No significant effects were found between conditions on this task (see Table 2).

**Table 2:** Mean percentages (and SDs) of “nearer” responses to left (cubes 1, 2, 3) or “upper” (cube 4) as a function of site (A, Experiment 1, Right PPC; B, Experiment 2, Left PPC), and TMS (Baseline, rTMS/Sham).

A. Experiment 1, right PPC					B. Experiment 2, left PPC			
	
Cube	Baseline	rTMS	Baseline	Sham	Baseline	rTMS	Baseline	Sham
1	55.88 (18.5)	61.08 (17.9)	61.35 (17.8)	61.54 (17.8)	66.92 (18.4)	63.06 (18.5)	63.44 (18.2)	60.58 (17.8)
2	45.86 (18.1)	40.96 (17.6)	37.69 (17.7)	36.35 (17.9)	31.41 (18.4)	33.59 (18.3)	33.08 (17.9)	36.15 (18.1)
3	50.96 (17.2)	44.81 (17.4)	42.88 (18.1)	42.31 (17.6)	57.69 (19)	55.00 (19.5)	65.70 (17.9)	60.00 (19.1)
Mean	50.90 (17.5)	48.59 (17.5)	47.31 (17.9)	46.73 (17.8)	52.01 (18.8)	50.55 (18.7)	54.07 (18.2)	52.24 (18.2)
4	47.88 (17.7)	45.77 (18.3)	46.54 (16.9)	41.92 (17.1)	33.08 (17.7)	33.01 (18.2)	32.43 (18.1)	32.76 (16.4)


### Experiment 2

#### Landmark Task

For the LT participants’ cumulative percentages of Task 1 + Task 2 are reported in Table 1 for the different conditions. Participants showed high accuracy (>86%) on the asymmetric stimuli, and no significant differences were found across conditions. In relation to the symmetrically bisected lines, participants’ behavior did not change with respect the baseline condition after rTMS over the left PPC. Indeed, they chose the right segment as longer similarly to the baseline (i.e., 48% in the baseline condition and 49.6% after Left rTMS), indicating that the inference with the Left PPC did not lead to a rightward bias. No significant effects were found for sham condition (see Table 1 and Figure 3B).

#### Necker Cube

The participants’ cumulative percentages for cubes 1, 2, and 3 and for cube 4, are reported in Table 2 for the different conditions. As for experiment 1, no significant effects were found between conditions on this task (see Table 2).

## Discussion

Prompted by earlier findings in patients with left-neglect (Bisiach et al., 1999), we investigated whether and how experimental manipulation of spatial attention by rTMS application to right PPC might affect Necker cubes perception in healthy participants. Correlations of lesion location to neglect symptoms has provided important knowledge on the neuroanatomy of spatial attention, but the extension of natural lesions posits a limit to drawing clear inferences on the specific function of the damaged area. By temporarily disrupting the activity of a focal region through TMS, it is possible to overcome this limitation (Ellison et al., 2004).

To verify the induction of a neglect-like bias after rTMS, participants were also tested on a line length estimation task (i.e., the Landmark Task, LT). Line length estimation is indeed a very sensitive and reliable task for the assessment of modulation of visuospatial attention (Learmonth et al., 2015) in neurological patients (Ricci et al., 2004, 2014, 2016; Savazzi et al., 2007; Chillemi et al., 2017a,b; Palermo et al., 2018) and healthy participants (Fierro et al., 2000; Ricci et al., 2012; Salatino et al., 2014).

In line with previous findings (Fierro et al., 2000; Ellison et al., 2004; Bjoertomt et al., 2009; Ricci et al., 2012; Salatino et al., 2014; Giglia et al., 2015; Giglhuber et al., 2017), the application of rTMS to right PPC induced significant neglect-like bias on the LT and no effects were found after rTMS of the left PPC or sham stimulation. Importantly, no significant effects were observed after active rTMS of right or left PPC on disambiguation of the NC. Although “absence of evidence is not evidence of absence,” the present findings seem to suggest that right PPC does not play a crucial role in the attentional dynamics putatively implicated in perception of the Necker cube (Bisiach et al., 1999). If as suggested by Bisiach et al. (1998) “the dynamics of spatial attention required for the disambiguation of visual patterns such as the Necker cube should be conceived in terms of a mental analog of three-dimensional navigation in real space rather than in terms of a beam of straight vectors leading directly from the viewer to configurational details,” then we might conclude that attentional processes governed by right PPC might not crucially be involved in illusory three-dimensional navigation. It is indeed very likely that the joint activity of other brain areas and/or bilateral parieto-frontal circuits (Chillemi et al., 2019) might better account for the complex attentional dynamics giving raise to illusory depth perception.

It is worth noticing that neglect syndrome has multi-faceted nature, as largely demonstrated by previous studies, reporting dissociations of neglect symptoms between tasks (Halligan and Marshall, 1992; Ferber and Karnath, 2001), and/or their presence in a variety of different tasks (see for example Robertson and Marshall, 1993; Bisiach and Vallar, 2000). The diverse nature of spatial neglect is supported by the present findings showing that right parietal rTMS selectively affected orientation of spatial attention on line length estimation, without affecting disambiguation of illusory three-dimensional objects. This outcome also excludes that the observed findings can be explained by generalized rTMS effects. Thus, in line with previous evidence, the present data support the importance of right PPC in the causation of neglect bisection bias and highlight that detection of neglect symptoms might largely dependent on the task used to assess the symptom.

The lack of significant effects on NC task may be due to different reasons. As previously discussed, it might be possible that our protocol did not target a critical brain region for illusory depth perception, that has been shown to be associated with bilateral activation of premotor and parietal areas (Inui et al., 2000). Perceptual processing of the Necker cube likely recruits more complex networks than those involved in LT (Ricci et al., 2012). Nonetheless, it is also possible that right PPC is somehow involved in NC perception but that the employed rTMS protocol was not optimal to modulate participants’ behavior. Although TMS pulses propagate along functionally connected areas (Ilmoniemi et al., 1997; Ricci et al., 2012), it is very unlikely that remote effects of offline rTMS were strong enough to disrupt cognitive processing in connected areas (Nikulin et al., 2003). Future studies, using online high-frequency rTMS might be necessary to probe crucial components of the above complex network. A limitation of the present study is that we tested a different group of participants in Experiment 2. This limitation was due to the number of the sessions composing each experiment (i.e., 9 sessions: Hunting procedure, 2 baselines, rTMS, and sham for the LT and 2 baselines, rTMS, and sham for the NC) and their duration. The time requested and the number of stimulation sessions made each experiment very demanding for the participants. Given this constrain and the aim of the study – i.e., within-subjects comparison of right PPC involvement on LT and NC processing - we decided to test a different group in experiment 2 (i.e., left PPC assessment).

To conclude, the present findings confirm that right PPC plays a crucial role for deployment of spatial attention on line length estimation in healthy participants. On the other hand, they suggest that this brain region is not critically involved in attentional disambiguation of three-dimensional Necker Cube. Future investigations employing optimal rTMS protocols, targeting different areas of fronto-parietal circuits, are necessary to clarify the neuro-functional bases of attentional contribution to illusory depth perception.

## Ethics Statement

This study was carried out in accordance with the recommendations of the Ethical Committee of the University of Turin, with written informed consent from all subjects. All subjects gave written informed consent in accordance with the Declaration of Helsinki. The protocol was approved by the Ethical Committee of the University of Turin.

## Author Contributions

AS and RR conceived and designed the study. AS, FG, and MPo acquired of the data. AS, FG, GC, MPy, and AB analyzed and interpreted of the data. AS and RR drafted the manuscript. All authors revised the manuscript critically for intellectual content and approved the final version to be published.

## Conflict of Interest Statement

The authors declare that the research was conducted in the absence of any commercial or financial relationships that could be construed as a potential conflict of interest.
